# Metabolomics analysis of the *lactobacillus plantarum* ATCC 14917 response to antibiotic stress

**DOI:** 10.1186/s12866-024-03385-3

**Published:** 2024-06-28

**Authors:** Yilin Zhong, Juan Guo, Yu Zheng, Huale Lin, Yubin Su

**Affiliations:** Department of Cell Biology & Institute of Biomedicine, MOE Key Laboratory of Tumor Molecular Biology, Guangdong Provincial Key Laboratory of Bioengineering Medicine, College of Life Science and Technology, National Engineering Research Center of Genetic Medicine, Jinan University, Guangzhou, 510632 China

**Keywords:** *Lactobacillus plantarum* ATCC14917, Metabolomics, Antibiotics, Probiotic protection

## Abstract

**Background:**

*Lactobacillus plantarum* has been found to play a significant role in maintaining the balance of intestinal flora in the human gut. However, it is sensitive to commonly used antibiotics and is often incidentally killed during treatment. We attempted to identify a means to protect *L. plantarum* ATCC14917 from the metabolic changes caused by two commonly used antibiotics, ampicillin, and doxycycline. We examined the metabolic changes under ampicillin and doxycycline treatment and assessed the protective effects of adding key exogenous metabolites.

**Results:**

Using metabolomics, we found that under the stress of ampicillin or doxycycline, *L. plantarum* ATCC14917 exhibited reduced metabolic activity, with purine metabolism a key metabolic pathway involved in this change. We then screened the key biomarkers in this metabolic pathway, guanine and adenosine diphosphate (ADP). The exogenous addition of each of these two metabolites significantly reduced the lethality of ampicillin and doxycycline on *L. plantarum* ATCC14917. Because purine metabolism is closely related to the production of reactive oxygen species (ROS), the results showed that the addition of guanine or ADP reduced intracellular ROS levels in *L. plantarum* ATCC14917. Moreover, the killing effects of ampicillin and doxycycline on *L. plantarum* ATCC14917 were restored by the addition of a ROS accelerator in the presence of guanine or ADP.

**Conclusions:**

The metabolic changes of *L. plantarum* ATCC14917 under antibiotic treatments were determined. Moreover, the metabolome information that was elucidated can be used to help *L. plantarum* cope with adverse stress, which will help probiotics become less vulnerable to antibiotics during clinical treatment.

**Supplementary Information:**

The online version contains supplementary material available at 10.1186/s12866-024-03385-3.

## Background

Lactic acid bacteria (LAB) are a group of Gram-positive fermentative bacteria, which are potentially beneficial for the gut ecosystem of humans [1]. In addition, LAB are often used as agents in food fermentation because they contribute to the preservation, flavour, and texture of fermented foods [2]. *Lactobacillus plantarum* are present in fermented foods and are also found in the human and other mammalian gut [3]. In addition to plant fermentation, *L. plantarum* has antioxidant, anti-inflammatory, anti-obesity, anti-diabetic, and anti-cancer properties [4]. *L. plantarum* H-6 can alleviate the diarrhoea caused by gut microbiota disorders after antibiotic treatment [5]. *L. plantarum* CCFM1143 alleviated diarrhoea by inflammation regulation and gut microbiota modulation [6]. *L. plantarum* WLPL2 decreased high cholesterol and hypercholesterolaemia [7], and *L. plantarum* JS19 as well as *L. plantarum* A3 prevented irritable bowel syndrome symptoms [8, 9]. Moreover, crude exopolysaccharides of *L. plantarum-12* significantly inhibited the proliferation of colon cancer HT-29 cells and thus showed potential anti-cancer activity [10].

Antibiotics are commonly used to treat bacterial infections. Studies have shown that most lactobacilli are sensitive to ampicillin and chloramphenicol [11, 12]. However, lactobacilli become resistant to tetracycline and erythromycin with the emergence of *erm*(B) and *tet*(M) genes [13, 14]. Among these antibiotics, ampicillin and tetracycline are commonly used in clinical treatment [15–18]. Ampicillin is a semisynthetic β-lactam antibiotic that has been used as a first-line treatment for a variety of infectious diseases. It acts during the active replication phase of bacteria by inhibiting bacterial cell wall synthesis [19–22]. Doxycycline is a synthetic antibiotic in the tetracycline class that is widely used. Doxycycline has a greater antibacterial efficacy than tetracycline [23]. However, frequent use at inappropriate doses and low mass concentrations can lead to the development of antibiotic resistance, which necessitates the use of higher doses to combat these resistant bacteria. In addition, the long-term ingestion of antibiotics can disrupt the balance of intestinal microflora, including probiotics [24].

*L. plantarum* has shown significant effects in regulating human health and has been confirmed to be a beneficial microorganism and also serves as a probiotic that adheres to intestinal epithelial cells or mucus [25]. However, when antibiotics are used clinically to treat pathogenic bacteria, the probiotics that colonise the intestines are often threatened. Therefore, it is difficult for intestinal probiotics to resist antibiotic stress under bacterial infection treatment. Many studies have shown that antibiotics can cause metabolic changes at the cellular level [26–28] and that metabolic perturbations can regulate bacterial sensitivity to antibiotics [29–31]. However, there are few reports on the use of metabolomics to study metabolic changes in *L. plantarum* under antibiotic stress.

*L. plantarum* ATCC14917 is a type strain of LAB; importantly, there are numerous studies showing that *L. plantarum* ATCC14917 exhibits probiotic activity [32–34]. The aim of this study was to investigate the metabolic changes in *L. plantarum* ATCC14917 by subjecting it to sublethal concentrations of ampicillin and doxycycline and subsequently analysing the intracellular metabolites using gas chromatography–mass spectrometry (GC–MS) metabolomics. We also investigated the key metabolic pathways and biomarkers involved in protecting *L. plantarum* from antibiotic killing effects. This study provides a theoretical basis for understanding how *L. plantarum* responds to antibiotic stress during the treatment of clinical bacterial infections.

## Materials and methods

### Bacterial strains, sources, culture conditions, and chemicals

*Lactobacillus plantarum* ATCC14917 used in this study was obtained from Professor Chunxi Peng, Jinan University. *L. plantarum* ATCC8014, *L. rhamnosus* ATCC53103, and *L. acidophilus* ATCC4356 were puarchased from HuanKai Microbiology Technology Co., Lid (Guangzhou, China). These strains were cultured at 37 °C for 18–24 h in de Man, Rogosa, and Sharp (MRS) broth (HuanKai Microbiology Technology Co., Ltd., Guangdong, China) with shaking at 220 rpm. Iso-Sensitest (IST) broth was purchased from Thermo Scientific (United States). LAB susceptibility test medium (LSM) was mixed from IST broth (90%) and MRS broth (10%), and adjusted to pH 6.7 [35]. Ampicillin, cefazolin, erythromycin, chloramphenicol, doxycycline, gentamicin, ciprofloxacin, and tobramycin were purchased from Sangon Biotech Co., Ltd. (Shanghai, China); guanine, adenosine diphosphate (ADP), nicotinamide, and triclosan were purchased from Macklin Biotech Co., Ltd. (Shanghai, China); and 2′,7′-dichlorodihydrofluorescein diacetate (DCFH-DA) was purchased from Sigma (United States).

### Measurement of minimum inhibitory concentration (MIC)

The MIC was determined by antimicrobial susceptibility testing, following a previously described method [36]. Briefly, bacterial cultures were grown in LSM for 18 h, then diluted 1:100 (v/v) in fresh LSM and inoculated at 37 °C, and grown to OD600 = 0.5. Bacterial cells (5 × 106 CFU/mL) were dispensed into each well of a 96-well polystyrene microplate containing two-fold serial dilutions of antibiotics. After 18 h of incubation, the minimal antibiotic concentrations that displayed no visible growth were determined as MIC values. Three biological replicates were used for each experiment.

### Determination of sublethal concentrations of ampicillin and doxycycline

The saturated bacterial cultures were pelleted by centrifugation for 5 min at 8,000 rpm. Then, the culture was diluted 1:100 in fresh MRS medium and cultured at 37 °C at 220 rpm with a concentration gradient of antibiotics for 12 h. After centrifugation at 8,000 rpm for 5 min, the samples were washed twice with PBS and resuspended in PBS to an OD600 of 1.0. Finally, 100 μL of the bacterial cultures was collected and serially diluted. A 10-μL aliquot of each culture was plated on MRS agar to determine the bacterial count [37].

### Metabolomics and data analysis

For sample preparation, a single bacterial colony was cultured in 30 mL of MRS broth for 18 h at 37 °C and 220 rpm. The saturated culture was then diluted 1:100 (v/v) in fresh MRS medium and incubated without or with 0.04 μg/mL ampicillin or 1.56 μg/mL doxycycline for 12 h. Each group was collected (control group without antibiotic treatments, ampicillin group, and doxycycline group), with six biological replicates for each group. Equal quantities of cells treated with antibiotics were collected at 8000 rpm for 5 min. The collected cells were quenched in liquid nitrogen to stop all metabolic processes, then cold methanol was added, and cells were stored at − 80 °C. Cells were lysed by sonication (200 W, 2-s pulse and 3-s pause, over ice, 5 min) to release more metabolites, and metabolites were extracted from the cell lysate using 1 mL of cold methanol containing 10 μg of ribitol as an internal standard [31]. The obtained samples were centrifuged at 12,000 g for 10 min at 4 °C, and the supernatant was dried using a rotary vacuum centrifuge. The dried extract was incubated with 50 μL of a methoxyamine hydrochloride solution (20 mg/mL, pyridine solution) for 1.5 h at 37 °C. Subsequently, 50 μL of N-methyl-N-(trimethylsilyl) trifluoroacetamide was added, and the solution was reacted for 1 h at 37 °C [38]. The purpose of this step was to allow all samples to undergo derivatization. The derivatized samples were centrifuged at 12,000 g for 15 min at 4 °C, the supernatant was removed, and the remaining pellet was used for subsequent experiments. Next, 1 μL of the derivative samples were used for gas chromatography-mass spectrometry (GC–MS) (GC–MS–QP 2010 plus, Shimadzu, Japan) analysis, and the experimental conditions for the inlet temperature, split ratio of the carrier gas (high-purity helium), and constant linear velocity were set to 300 °C, 5:1, and 40.0 cm/s, respectively [39]. Metabolites were separated using a DB-5 MS capillary column (30 m × 250 μm × 0.25 μm, J&W Scientific Inc., USA). Mass signals of metabolites were obtained using a full scan mode. Finally, by analysing a light diesel sample with the same instrumental parameters as above, through this step, the retention index (equivalent to the retention time of n-alkanes) of the bacterial metabolites was obtained.

Peak processing was performed using the XCMS method on raw mass spectrometry data (NetCDF format) exported by GC–MS Solution 4.2 (Shimadzu, Japan) [40]. Metabolites were first identified by searching against commercial mass spectral libraries and then further validated against available standards. Statistical analysis of data was performed using IBM SPSS Statistics (version 22.0; SPSS Inc., Chicago, IL, USA), and data with a p value less than 0.05 were considered to have significant differences. In this process, the ampicillin group and the doxycycline group were compared with the control group to identify differential metabolites, which were used for subsequent analysis. Hierarchical clustering analysis was implemented through R Studio software (version 4.0.3). Principal component analysis and orthogonal partial least squares analyses were performed using SIMCA–P + . Z-score analysis was performed in Microsoft Excel by determining differences of means. Metabolic pathway enrichment was performed using MetaboAnalyst 5.0. Graphs were drawn using Microsoft Excel and GraphPad Prism 8.0 (San Diego, CA, USA) [41].

### Antibiotic bactericidal assays

Antibiotic bactericidal assay was performed as described previously [38]. The only difference was that the M9 minimal was replaced with MRS broth. Briefly, a single colony was picked, and grown in 30 mL MRS broth in 100 mL flasks at 37 °C with shaking at 220 rpm for 18 h. After centrifugation for 5 min at 8,000 rpm to remove the supernatant, and was washed twice with sterile saline and then re-suspended in fresh MRS medium to an OD600 of 0.2. The cells were treated with guanine and ADP with ampicillin or doxycycline at 37℃, 220 rpm for 6 h. Finally, 100 μL of the bacterial cultures were collected and serially diluted. An aliquot of 10 μL each culture was plated on MRS agar to determine the bacterial count.

### Measurement of ROS

Intracellular ROS generation was measured as described [42]. Briefly, saturated bacterial cultures were pelleted by centrifugation at 8,000 rpm for 5 min. The samples were washed twice by sterile saline and re-suspended in fresh MRS medium to an OD600 of 0.2. Antibiotics (0.156 μg/mL ampicillin or 6.25 μg/mL doxycycline) or/and metabolites (2 mM guanine or 20 mM ADP) were added to the medium and incubated at 37 °C at 220 rpm for 6 h. Then, equivalent amounts of bacteria were collected by centrifugation at 8,000 rpm for 5 min and washed three times with PBS. Cells were re-suspended with 500 μL PBS and sonication (200-W total power with 50% output, 2-s pulse, and 3-s pause over ice) for 6 min, and cellular debris was removed by centrifugation at 8,000 rpm for 5 min. Cell-free supernatants were stained with DCFH-DA (Sigma, United States). Then, 150 μL of the cell lysates were added to each well in a 96-well plate, followed by addition of 10 μL H_2_DCFDA and PBS to achieve final volume of 200 μL, which was incubated for 30 min at 37 °C in the dark. Fluorescence was immediately measured using the fluorescence plate reader (Biotek, Synergy HT, Vermont, United States) at an excitation wavelength of 485 nm and an emission wavelength of 515 nm.

## Results

### Sensitivity of *L. plantarum* ATCC14917 to antibiotics

**Fig. 1:**
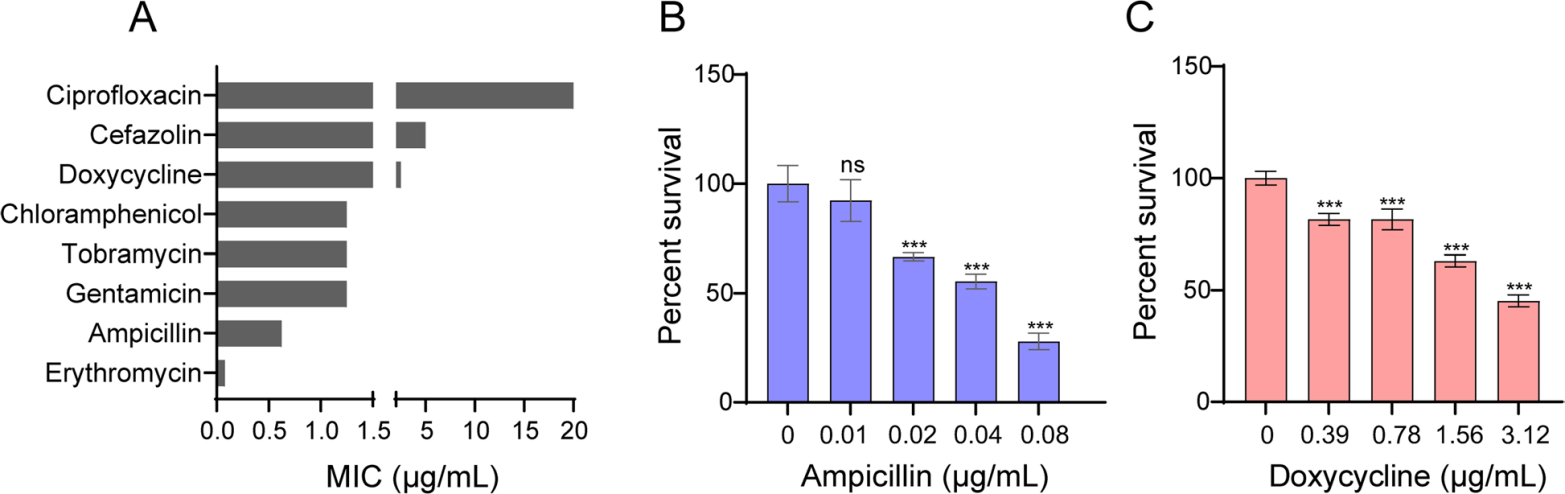
MIC determination and determination of sublethal concentrations of ampicilin and doxycyline. **A** MIC of *L. plantarum* ATCC14917 to different types of antibiotics. **B** Survival of *L. plantarum* ATCC14917 at different concentrations of ampicillin. **C** Survival of *L. plantarum* ATCC14917 at different concentrations of doxycycline. All data are displayed as mean ± SEM. ns: non-significant differences. ***p < 0.001, determined by Student’s t test

We selected eight different antibiotics used in the clinical treatment of microbial infections [43, 44] and measured their MICs to determine the sensitivity of *L. plantarum* ATCC14917 to different antibiotics [45]. From the results **(**Fig. 1A), the MICs of β-lactams (ampicillin = 0.625 μg/mL, cefazolin = 5 μg/mL), a macrolide (erythromycin = 0.07825 μg/mL), a chloramphenicol (chloramphenicol = 1.25 μg/mL), a tetracycline (doxycycline = 2.5 μg/mL), and aminoglycosides (gentamicin = 1.25 μg/mL, tobramycin = 0.625 μg/mL) were all lower than or equal to 5 μg/mL, indicating that *L. plantarum* ATCC14917 was sensitive to these seven antibiotics. However, the strain was less sensitive to ciprofloxacin. Based on statistics on clinically used antibiotics for the treatment of common microbial infections in recent years, ampicillin and doxycycline were selected for further experiments [46–50].

In many reports, sublethal concentrations of antibiotics have been used to stress bacteria to observe the induced physiological changes [51–53]. In this study, antibiotics at different concentrations were used to define sublethal concentrations. The results are shown in Fig. 1B and C. The viability of *L. plantarum* ATCC14917 was reduced with increasing doses of ampicillin and doxycycline. At a concentration of 0.04 μg/mL ampicillin or 1.56 μg/mL doxycycline, survival rates of *L. plantarum* ATCC14917 decreased approximately 50%. Therefore, these two antibiotic concentrations were considered sublethal and were selected to induce stress in *L. plantarum* ATCC14917.

### Significant changes in the bacterial metabolome under ampicillin or doxycycline stress

To explore changes in the metabolic state of bacteria under sublethal concentrations of antibiotics, GC–MS was used to analyse the cell metabolome in the presence or absence of ampicillin and doxycycline. The antibiotic-unstressed and antibiotic-stressed groups showed different metabolic profiles. A global metabolite heat map was obtained by inputting the data into R software and analysing the metabolite changes in each sample. Supplementary Fig. S1A and B show the 88 identified metabolites. All metabolites were divided into five categories: amino acids, carbohydrates, nucleotides, fatty acids and lipids, and others. The proportions were 39.77% amino acids, 25% carbohydrates, 21.59% nucleotides, 10.23% fatty acids and lipids, and 3.41% others (Supplementary Fig. S1C).

**Fig. 2:**
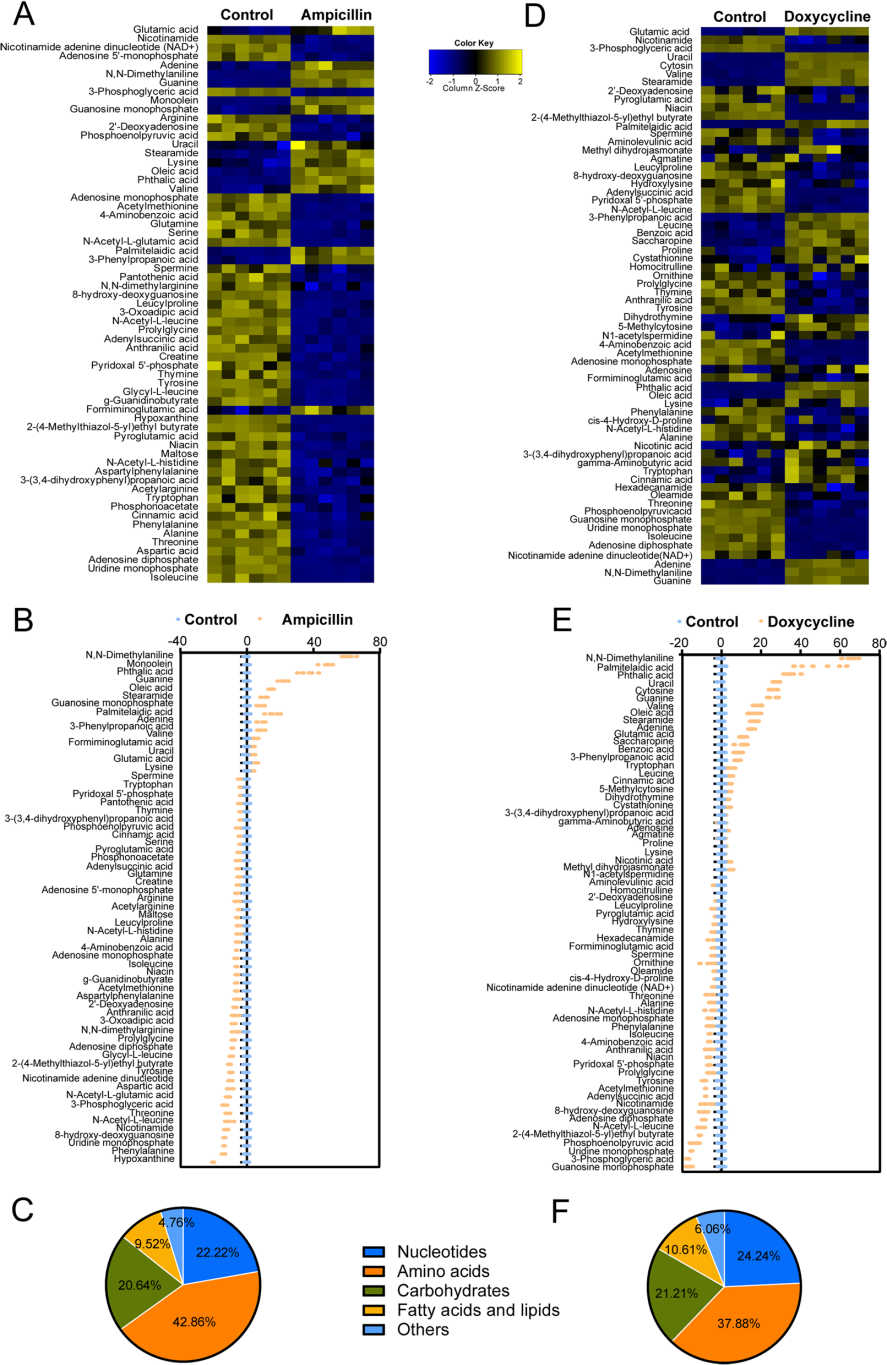
Changes in differential metabolites of *L. plantarum* ATCC14917 in response to ampicillin and doxycycline. **A** & **C** Heat map showing differential abundance of metabolites. Blue and yellow colors indicate lower and higher abundances of the metabolites relative to the mean level of the control group, respectively. **B** & **D** Z-score plots of changes in differential metabolites based on control. The data were respectively scaled to the mean and standard deviation of control. Each point represents one biological repeat. Different treatments are distinguished by the color. **E** & **F** Categories of differential abundance of different metabolites

To better understand the effects of ampicillin or doxycycline on *L. plantarum* ATCC14917, IBM SPSS Statistics 22 was used to conduct a non-parametric test (Mann–Whitney U test) on the standardised data, and these data were used to obtain heat maps and Z-score plots. Compared with the control group, a total of 63 metabolites showed differential abundances (*P* < 0.05) with ampicillin treatment (Fig. 2A). Of these metabolites, 48 were present at a lower abundance, and 15 were present at a higher abundance in the presence of ampicillin (Fig. 2B). Among these, N-acetyl-L-leucine, 8-hydroxy-deoxyguanosine, uridine monophosphate, phenylalanine, and hypoxanthine were among the top five downregulated metabolites, whereas N, N-dimethylanilin, monoolein, phthalic acid, guanine, and oleic acid were the top five upregulated metabolites. Among these metabolites, the proportions included 42.86% amino acids, 20.64% carbohydrates, 22.22% nucleotides, 9.52% fatty acids and lipids, and 4.76% others (Fig. 2C). The same analysis revealed that 66 metabolites were differentially expressed (*P* < 0.05) between doxycycline and the control group **(**Fig. 2D). Of these metabolites, 38 were present at a lower abundance in the presence of doxycycline, and 28 were present at a higher abundance (Fig. 2E). Among these, 2-(4-methylthiazol-5-yl)ethyl butyrate, phosphoenolpyruvic acid, uridine monophosphate, 3-phosphoglyceric acid, and guanosine monophosphate were the top five downregulated metabolites, whereas N, N-dimethylaniline, palmitelaidic acid, phthalic acid, uracil, and cytosine were the top five upregulated metabolites. Among these metabolites, the proportions were 37.88% amino acids, 24.24% nucleotides, 21.21% carbohydrates, 10.61% fatty acids and lipids, and 6.06% others (Fig. 2F). These results indicated that most metabolites in bacteria are downregulated under antibiotic stress.

### Purine metabolism changes significantly in *bacteria* under antibiotic stress

**Fig. 3:**
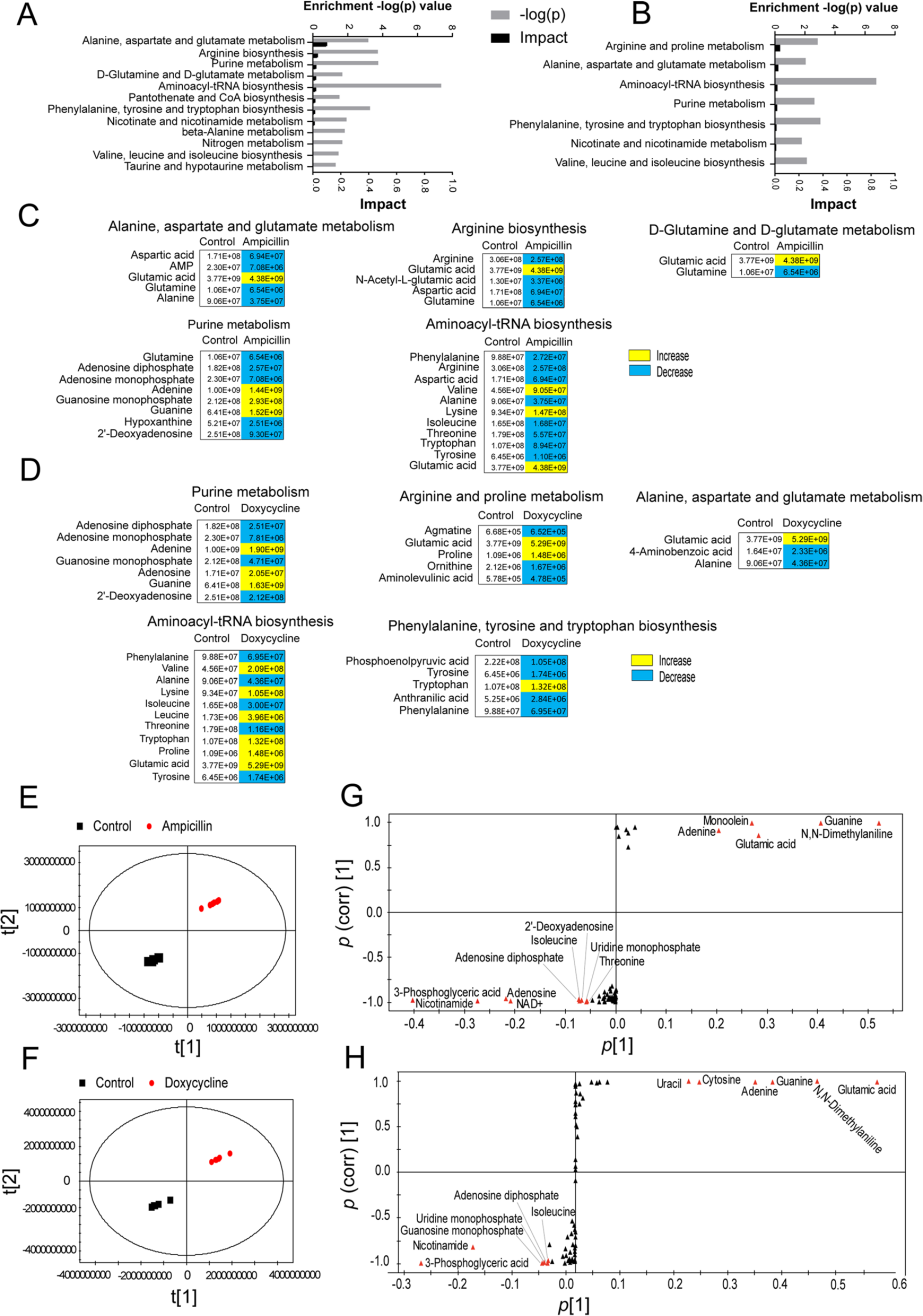
Enrichment of metabolic pathways in *L. plantarum* ATCC14917 in response to ampicillin and doxycycline. **A** & **B** Significantly enriched metabolic pathways in response to uracil treatment (*p* < 0.05). **C** & **D** Changes in differential metabolites involved in the significantly enriched pathways. Yellow color and blue color indicate increased and decreased metabolites, respectively, in ampicillin-treated and doxycycline-treated group. E & F: The score plot of PCA. Each dot represents one biological replica in the plot. G & H: S-plot of OPLS-DA. Triangle represents individual metabolite. Red indicates the potential biomarkers, which is greater than or equal to 0.05 and 0.5 for absolute value of covariance *p* [1] and correlation *p* (corr) [1], respectively

Most metabolites are associated with metabolic pathways, and different metabolic pathways are interconnected to form a complicated metabolic network. Pathway enrichment was performed by inputting differential metabolites into the online website application MetaboAnalyst 6.0. The results are shown in Fig. 3A. In comparison with the control, a total of 12 metabolic pathways were enriched for differential metabolites under ampicillin treatment, while seven metabolic pathways were enriched under doxycycline treatment (Fig. 3B). Ampicillin- and doxycycline-treated cells shared alanine, aspartate, and glutamate metabolism; aminoacyl-tRNA biosynthesis; purine metabolism; nicotinate and nicotinamide metabolism; phenylalanine, tyrosine, and tryptophan biosynthesis; and valine, leucine, and isoleucine biosynthesis. Pathway enrichment analysis revealed that purine metabolism was among the top four enriched pathways, demonstrating its importance in metabolic regulation.

We further analysed the changes in the differential metabolites of the enriched metabolic pathways. As shown in Fig. 3C and D, in purine metabolism, most of the metabolites decreased under ampicillin and doxycycline treatment compared with the control, including adenosine diphosphate (ADP), adenosine monophosphate, adenine, guanosine monophosphate, 2’-deoxyadenosine, and adenosine. Principal component analysis was applied to identify the components, in which component t[1] distinguished *L. plantarum* ATCC14917 from exposure to ampicillin or doxycycline (Fig. 3E and F). The discriminating variables are presented as S-plots. The cut-off value for the absolute value of covariance p[1] was ≥ 0.05 and for correlation p (corr) was ≥ 0.5. The S-plot was plotted from the predicted components p[1] vs. p (corr), representing the magnitude (modelled covariation) and reliability (modelled correlation), respectively. The selection of potentially interesting biochemical compounds required the combination of covariance and correlation, which was visualized by an S-plot.

When comparing metabolites of *L. plantarum* ATCC14917 with and without ampicillin treatment, we found a total of 14 potential biomarkers, among which five metabolites were upregulated: adenine, glutamic acid, guanine, monoolein, and N, N-dimethylaniline; and nine metabolites were downregulated: nicotinamide, 3-phosphoglyceric acid, ADP, adenosine, nicotinamide adenine dinucleotide (NAD^+^), 2'-deoxyadenosine, uridine monophosphate (UMP), threonine, and isoleucine (Fig. 3G and Supplementary Fig. 2A and B). There was a total of 12 biomarkers in the doxycycline-treated group, among which cytosine, adenine, N, N-dimethylaniline, glutamic acid, uracil, and guanine were upregulated, whereas 3-phosphoglyceric acid, UMP, isoleucine, nicotinamide, ADP, and guanosine monophosphate were downregulated (Fig. 3H and Supplementary Fig. 2C and D). We found that the biomarkers shared by these two groups—adenine, guanine, N, N-dimethylaniline, nicotinamide, adenosine, 3-phosphoglyceric acid, ADP, and UMP—were involved in purine metabolism. These results indicate that antibiotic stress in *L. plantarum* ATCC14917 affects purine metabolism.

### Promoting purine metabolism helps *Lactobacillus* respond to antibiotic stress

**Fig. 4:**
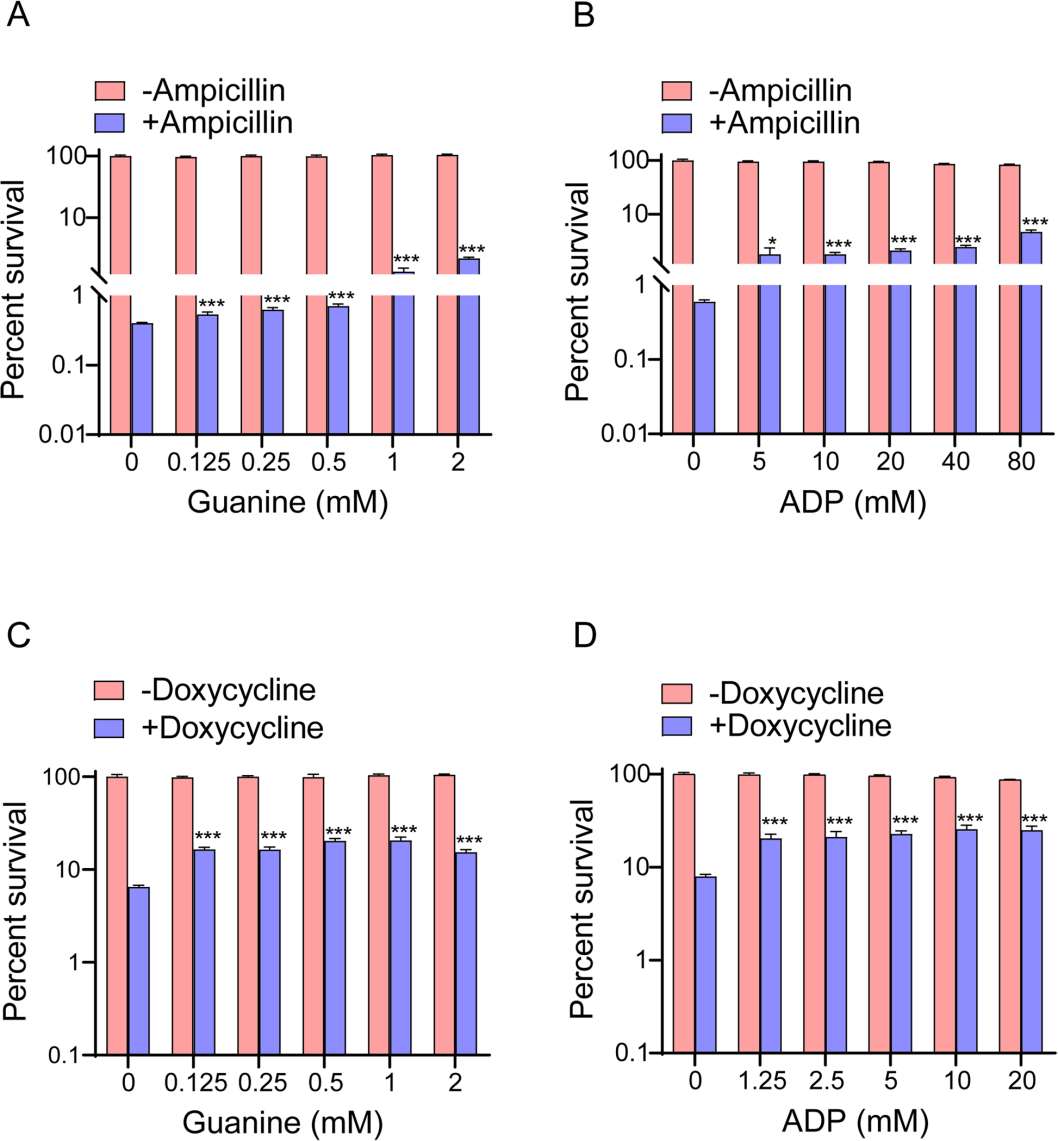
Effect of ADP, guanine cooperate with ampicillin and doxycycline on *L. plantarum* ATCC14917 survival.  **A** and **B**  The killing effects of different concentrations of ADP and guanine combined with 0.156 μg/mL ampicillin. **C** and **D** The killing effects of different concentrations of ADP and guanine combined with 6.25 μg/mL doxycycline. All data are displayed as mean ± SEM. *p < 0.05 and ***p < 0.001, determined by Student’s t test

The analysis of potential biomarkers and pathway enrichment showed that purine metabolism might be closely related to changes in *L. plantarum* ATCC14917 after antibiotic stress. It was hypothesised that the exogenous administration of purine supplements (such as guanine or ADP) that regulate purine metabolism may help *L. plantarum* ATCC14917 cope with antibiotic stress and improve its survival rate. To test this hypothesis, we compared the viability of *L. plantarum* ATCC14917 under ampicillin stress with and without the addition of guanine or ADP. The results showed that at the indicated ampicillin concentrations, cell survival was reduced (Supplementary Fig. 3A). In the presence of 0.156 μg/mL ampicillin, about 99% of the bacterial cells were killed, but after adding guanine, the bacterial survival rate increased by approximately five-fold (Fig. 4A). After the addition of ADP, the bacterial survival rate increased by about eight-fold (Fig. 4B). Similarly, we also compared the bacterial viability of *L. plantarum* ATCC14917 under doxycycline stress with or without the addition of guanine or ADP. The viability of *L. plantarum* ATCC14917 was reduced with various doses of doxycycline (Supplementary Fig. 3B). Approximately 95% of bacterial cells were killed using 6.25 μg/mL doxycycline alone, but after adding guanine, the bacterial survival rate increased by approximately two-fold (Fig. 4C); after adding ADP, the bacterial survival rate increased by approximately three-fold (Fig. 4D). These results show that guanine and ADP can reduce the lethality of *L. plantarum* ATCC14917 caused by ampicillin and doxycycline in a dose-dependent manner. To explore the general effects of guanine and ADP on lactic acid bacteria, experiments were also conducted on *L. plantarum* ATCC8014, *L. acidophilus* ATCC4356, and *L. rhamnosus* ATCC53103. The results showed that guanine and ADP provided strong protection of *L. plantarum* ATCC8014 and *L. acidophilus* ATCC4356 from ampicillin and doxycycline (Supplementary Fig. 4A–D). However, as for *L. rhamnosus* ATCC53103, only guanine reduced the damage caused by doxycycline and ampicillin. ADP alleviated damage caused by doxycycline alone (Supplementary Fig. 4E) but was ineffective against ampicillin (Supplementary Fig. 4F).

### Addition of exogenous metabolites can reduce bacterial intracellular ROS levels

**Fig. 5:**
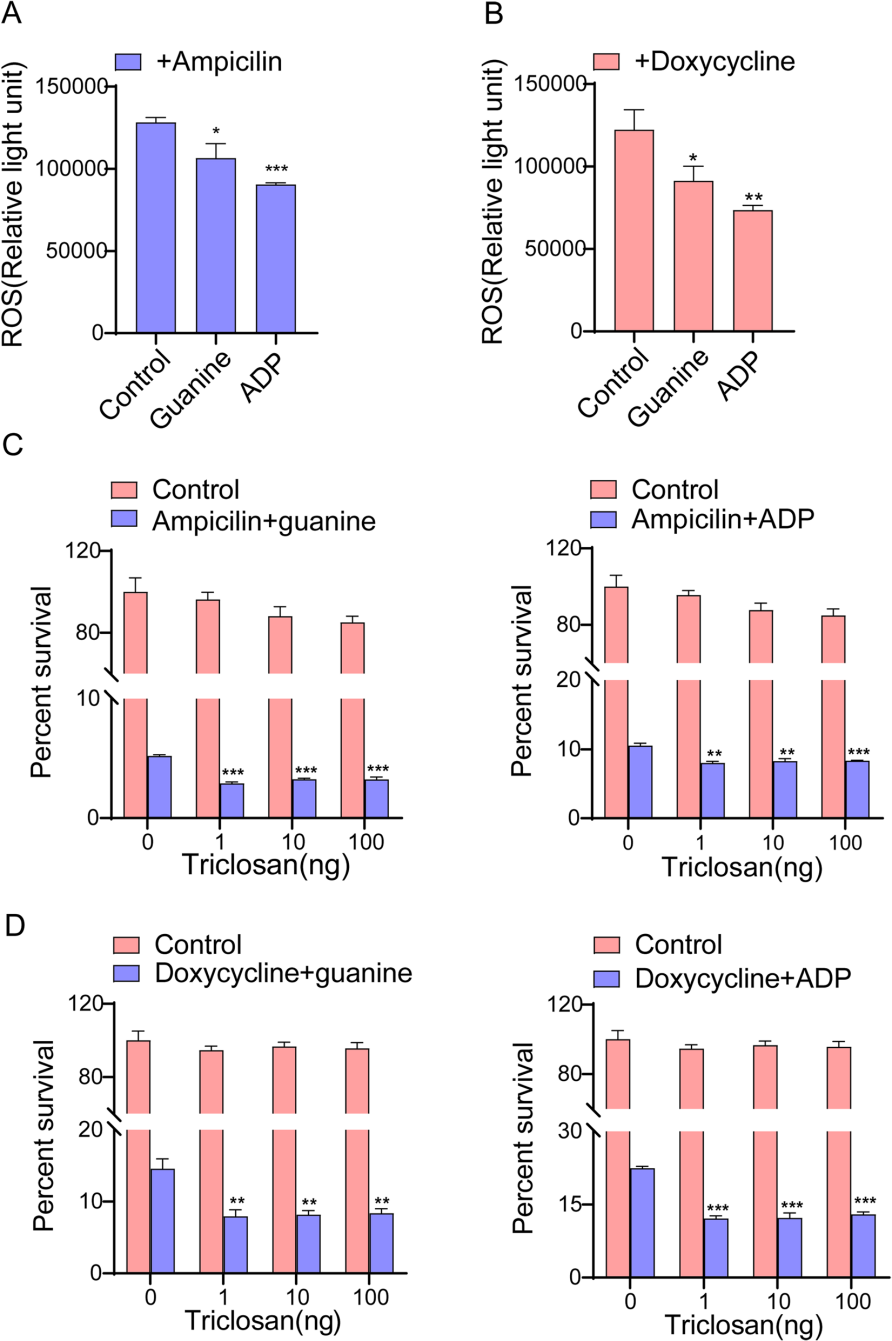
Effects of guanine, ADP cooperating with ampicillin, doxycycline on ROS of *L. plantarum* ATCC14917. **A** & **B** Effects of guanine, ADP cooperating with ampicillin, doxycycline on ROS content. **C** & **D** Effect of guanine, ADP cooperating with ampicillin, doxycycline on *L. plantarum* ATCC14917 survival at different doses of trichloride. All data are displayed as mean ± SEM. **p < 0.01 and ***p < 0.001, determined by Student’s t test

Although the primary mechanism of ampicillin lethality is inhibiting cell wall synthesis, and of doxycycline is inhibition of protein synthesis [54, 55], this study mainly investigated metabolic aspects of the effects of antibiotics, to explore how metabolites affect oxidative stress with antibiotic treatment. To understand how guanine and ADP protect bacteria against antibiotic damage, intracellular ROS levels were evaluated. ROS is generally considered to include superoxide, hydrogen peroxide, and hydroxyl radicals [56, 57], and the accumulation of ROS often leads to bacteria death. The results revealed that ampicillin and doxycycline increased intracellular ROS levels, and guanine and ADP eliminated the increased ROS levels caused by antibiotics (Fig. 5A and B). To further verify the mechanism of how guanine and ADP improved *L. plantarum* ATCC14917 activity against ROS, triclosan was used to promote intracellular production of ROS [58, 59]. The results showed that after the addition of triclosan, survival rates of *L. plantarum* ATCC14917 decreased by approximately two-fold in the presence of metabolites and antibiotics (Fig. 5C and D). These results show that purine metabolism affects the production of ROS and thus the killing effect of antibiotics on *L. plantarum* ATCC14917.

## Discussion

LAB are often used in food fermentation due to their unique properties. Moreover, most lactic acid bacteria, such as *L. plantarum* ATCC14917, *L. plantarum* CCFM1143, and *L. plantarum* H-6, exhibit probiotic activity, which can have a positive impact on host health. However, alterations in the composition of the human intestinal microbiome may contribute to dysbiosis, which is associated with gastrointestinal side effects during anti-cancer treatment, antibiotic administration, and exposure to infectious agents [60]. In particular, with the increase in drug-resistant bacteria, high doses of antibiotics have posed a tremendous threat to LAB in the human body. Therefore, it is necessary to find suitable methods to protect *L. plantarum* and other LAB from antibiotics.

When bacteria are under adverse environmental stress, they adjust the environment within the cell to resist the adverse factors. Understanding these intracellular changes can help us develop new strategies to protect *L. plantarum*. Previously, studies have used transcriptome, metabolome, and proteome methods to reveal changes that occurred within *L. plantarum* or other LAB under stress. Mbye et al. used proteomics to explain that lactic acid bacteria respond to different environmental stresses through unique protein expression strategies [61]; Huang et al. and Pieteres et al. used transcriptomics to show intracellular changes in *L. plantarum* facing acid–base stress [62, 63]; Wang et al. and Wu et al. used metabolomics to elucidate the response of *L. plantarum* under acid–base stress [64, 65]. These studies have helped us to better understand changes that occur within *L. plantarum* in times of adversity, and also provide a theoretical basis for protecting *L. plantarum*. However, there are few studies focusing on changes in *L. plantarum* in response to antibiotic pressure.

This study used metabolomics to reveal metabolic changes that occur in *L. plantarum* ATCC14917 under stress induced by sublethal concentrations of ampicillin or doxycycline. Compared with the control group, metabolic pathways related to amino acid, protein, and nucleotide synthesis were downregulated after antibiotic stress, including alanine, aspartate, and glutamate metabolism; aminoacyl-tRNA biosynthesis; and purine metabolism, which were highly ranked in pathway enrichment. Alanine, aspartate, and glutamate metabolism, which participate in energy metabolism in the cell and the metabolic cycle of amino acids, were downregulated. Amino acids serve as the fundamental building blocks of proteins and play a crucial role as primary carriers in life-sustaining activities. Aminoacyl-tRNA biosynthesis is related to intracellular protein synthesis, and purine metabolism regulates the synthesis and decomposition of the purines involved in nucleotide biosynthesis. Therefore, these key metabolic pathways may indicate that, when faced with stress by antibiotics, *L. plantarum* will resist adverse environments by regulating its own energy and physical activities.

Further analysis identified eight biomarkers involved in purine metabolism when guanine and ADP were present in both the ampicillin and doxycycline treatment groups. Addition of exogenous guanine and ADP protected *L. plantarum* from damage by antibiotics and thus improved the survival rate. We speculated that the protective effect was related to activated purine metabolism. Purine metabolism is an important metabolic pathway in bacteria, and previous studies have shown that defects in purine metabolism render cells more susceptible to oxidative bursts [66, 67] and can change their sensitivity to antibiotics [68]. There are also many studies showing that when *L. plantarum* was subjected to oxidative stress, purine metabolism changed [69–71]. The primary mechanism of ampicillin is inhibition of cell wall synthesis, and that of doxycycline is inhibition of protein synthesis. Both antibiotics can also lead to bacterial death by increasing intracellular ROS levels [72–75]. In further experiments, it was found that guanine and ADP reduced the ROS level increase caused by ampicillin and doxycycline. It can be concluded that activation of purine metabolism helped resist damage caused by reactive oxygen species and improved the survival rate of *L. plantarum* ATCC14917. We further verified this using ROS promoters (such as triclosan) in subsequent experiments. After the addition of triclosan, the protective effects of guanine and ADP on bacteria were weakened.

When *L. plantarum* ATCC14917 was stressed by antibiotics, most pathways were downregulated, among which purine metabolism was most clearly downregulated. By exogenously adding the downregulated purine metabolism intermediates guanine and ADP, *L. plantarum* ATCC14917 was protected from antibiotics and exhibited an improved survival rate. Guanine and ADP activated purine metabolism and effectively reduced high ROS levels caused by ampicillin and doxycycline. However, this study did not elucidate the mechanism by which purine metabolism reduces intracellular ROS levels, which merits further study.

## Conclusion

In the present study, we found that the use of ampicillin or doxycycline to stress *L. plantarum* ATCC14917 had a significant impact on its metabolism. After exposure to antibiotics, *L. plantarum* ATCC14917 appeared to protect itself from damage by altering its metabolic activity. Therefore, we speculated that it may be possible to protect *L. plantarum* ATCC14917 from antibiotics by regulating its metabolism. Exogenous addition of biomarkers involved in purine metabolism had a marked effect on survival, and guanine and ADP eliminated ROS induced by ampicillin or doxycycline. These results deepen our understanding of the self-regulatory mechanism of *L. plantarum* ATCC14917 under antibiotic stress and also provide potential strategies for protecting *L. plantarum* and other gut probiotics during antibiotic treatment. Recently studies confirmed that metabolite activation of purine metabolism promotes the killing effect of antibiotics or serum against other pathogenic bacteria [68, 76, 77]. This study found that the metabolites guanine and ADP used together with antibiotics for treating clinical infections may protect LAB from antibiotic damage and may also effectively eradicate pathogenic bacteria in humans.

### Supplementary Information

Supplementary Material 1.

Supplementary Material 2.

Supplementary Material 3.

Supplementary Material 4.

Supplementary Material 5.

## Data Availability

Data and materials will be made available upon request.
