# Supplementary figures and images for: Metabolomics analysis of the lactobacillus plantarum ATCC 14917 response to antibiotic stress
Source: BMC Microbiol. 2024 Jun 28;24:229. doi: 10.1186/s12866-024-03385-3 (PMC11212188; doi:10.1186/s12866-024-03385-3)

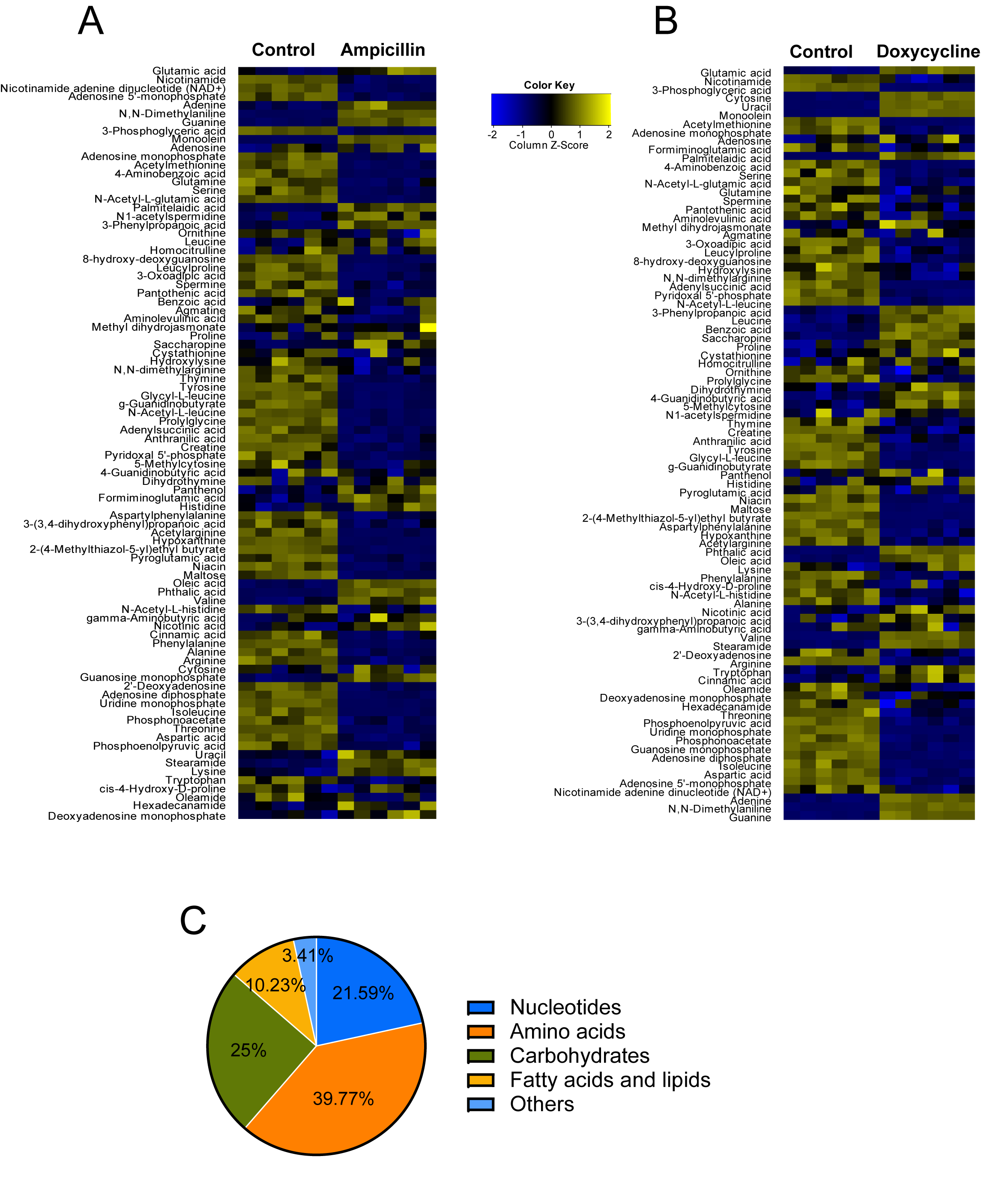

Supplement: Supplementary file 1 — Supplementary Material 1. [file 12866_2024_3385_MOESM1_ESM.tif]

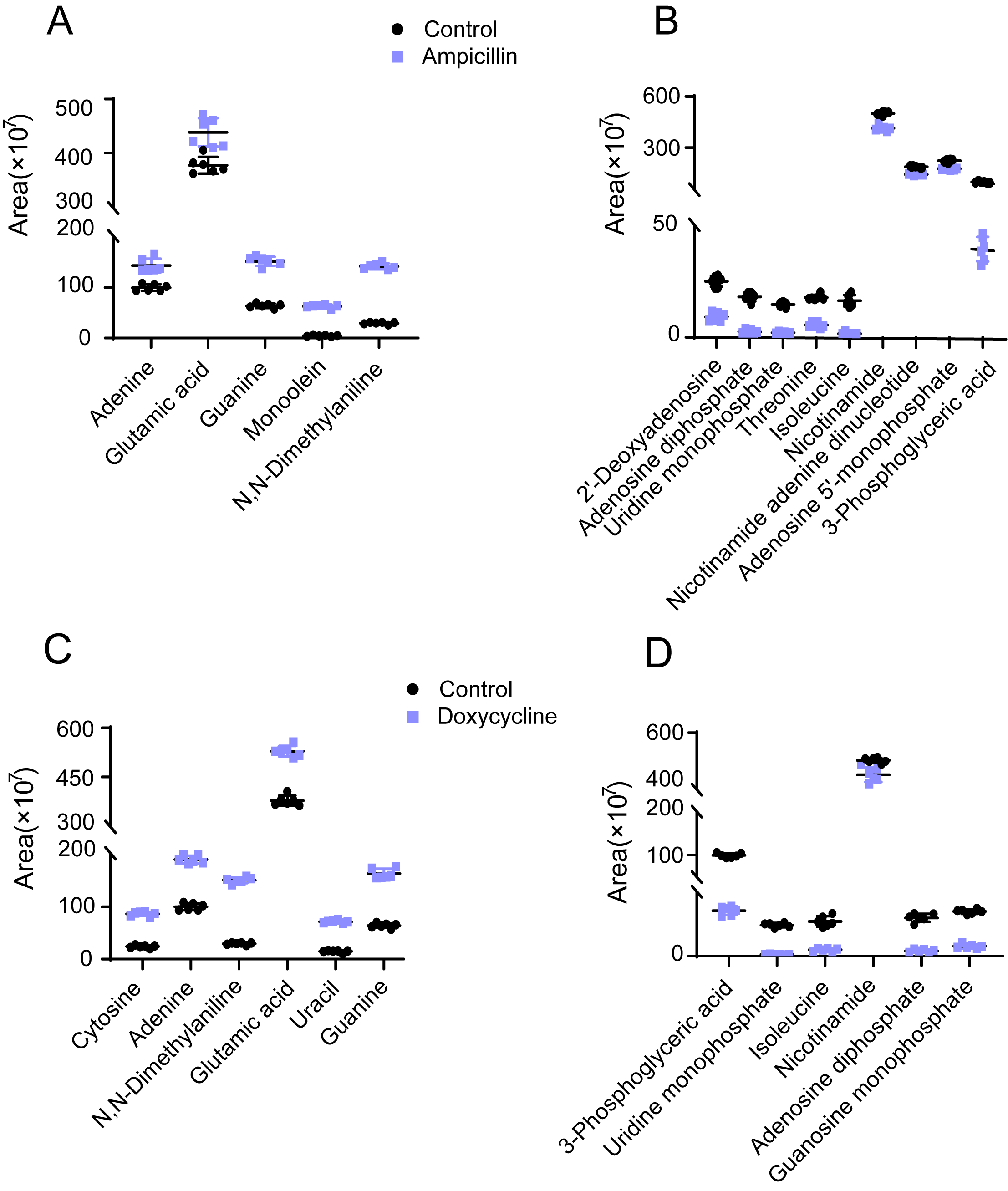

Supplement: Supplementary file 2 — Supplementary Material 2. [file 12866_2024_3385_MOESM2_ESM.tif]

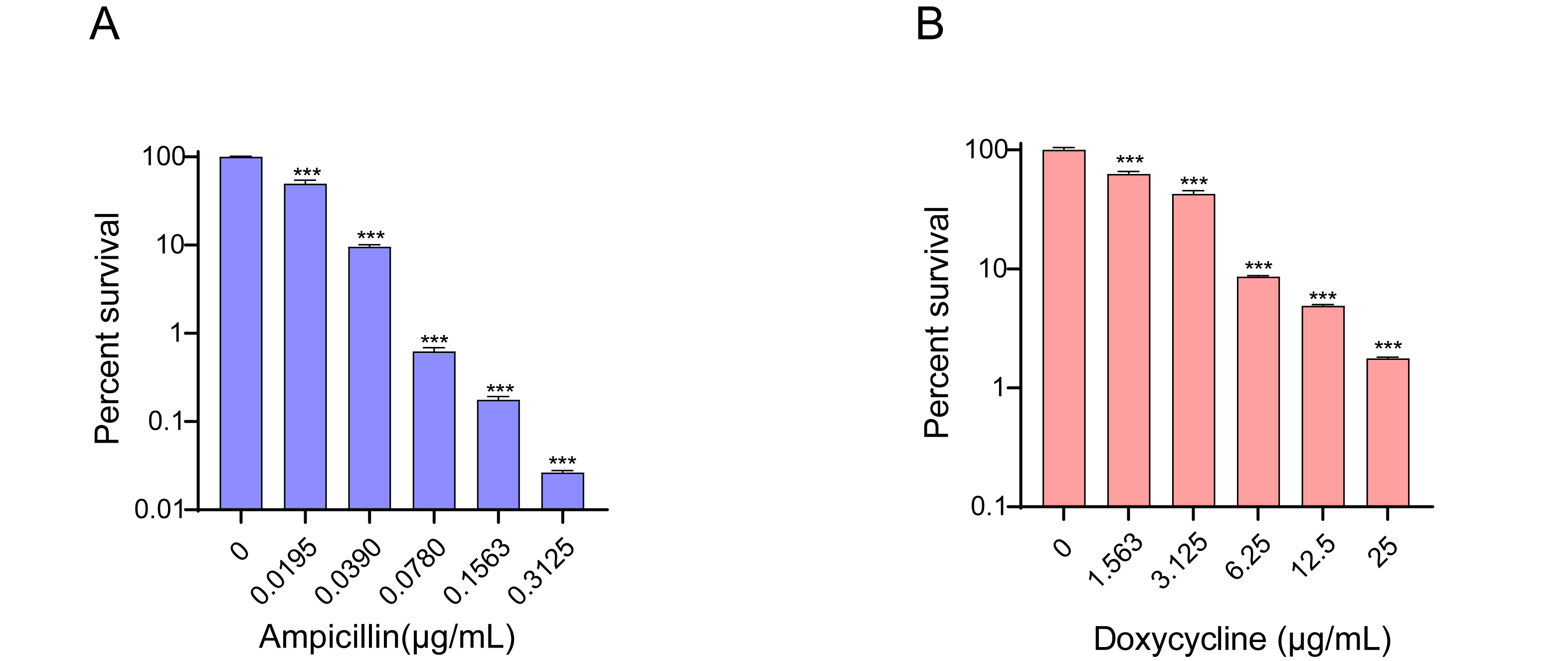

Supplement: Supplementary file 3 — Supplementary Material 3. [file 12866_2024_3385_MOESM3_ESM.tif]

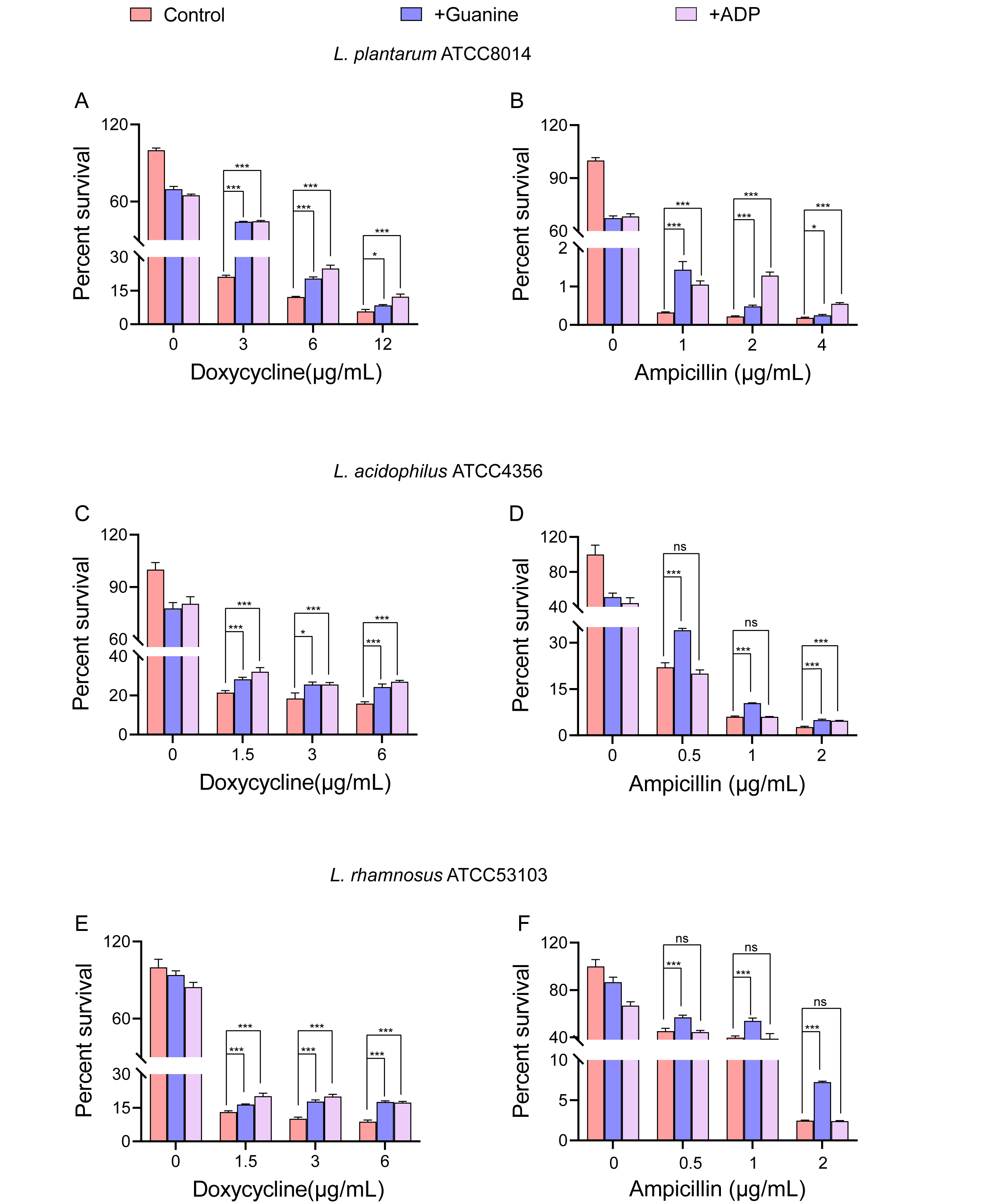

Supplement: Supplementary file 4 — Supplementary Material 4. [file 12866_2024_3385_MOESM4_ESM.tif]
