# Supplementary material for: Metabolomics analysis of the lactobacillus plantarum ATCC 14917 response to antibiotic stress
Source: BMC Microbiol. 2024 Jun 28;24:229. doi: 10.1186/s12866-024-03385-3 (PMC11212188; doi:10.1186/s12866-024-03385-3)
Supplement: Supplementary file 5 — Supplementary Material 5. [file 12866_2024_3385_MOESM5_ESM.docx]

**Supplementary Fig. 1** Changes in differential metabolites of *L. plantarum* ATCC14917 in response to ampicillin and doxycycline. A & B: Heat map showing differential abundance of all metabolites between control and ampicillin or doxycycline. Blue and yellow colors indicate lower and higher abundances of the metabolites relative to the mean level of the control group, respectively. C: Categories of differential abundance of all metabolites.

**Supplementary Fig. 2** The potential biomarkers of ampicillin and doxycycline. A & B: The scatter plot of biomarkers in data of ampicillin. Compared with the control, abundance of metabolites are up-regulated (A) and down-regulated (B) in the ampicillin-treated group. C & D: The scatter plot of biomarkers in data of doxycycline. Compared with the control, abundance of metabolites are up-regulated (C) and down-regulated (D) in the doxycycline-treated group.

**Supplementary Fig. 3** Antibiotic concentration screening of *L. plantarum* ATCC14917 in antibiotic bactericidal assays. A & B : Survival of *L. plantarum* ATCC14917 at different concentrations of ampicillin (A) or doxycycline (B). All data are displayed as mean ± SEM. ****p* < 0.001, determined by Student’s t test.

**Supplementary Fig. 4** Effect of ADP, guanine cooperate with ampicillin and doxycycline on survival of LAB. A & C & E : The concentration effect of doxycycline combined with 1mM guanine or 10mM ADP on survival rate of *L. plantarum* ATCC8014, *L. acidophilus* ATCC4356 and *L. rhamnosus* ATCC53103. B & D & F: The concentration effect of ampicillin combined with 2mM guanine or 80mM ADP on survival rate of *L. plantarum* ATCC8014, *L. acidophilus* ATCC4356 and *L. rhamnosus* ATCC53103. All data are displayed as mean ± SEM. ns indicates no significance, **p* < 0.05 and ****p* < 0.001, determined by Student’s t test.
